# A fluid model of pulsed direct current planar magnetron discharge

**DOI:** 10.1038/s41598-023-36231-z

**Published:** 2023-06-03

**Authors:** Si Bui Quang Tran, Fong Yew Leong, Ramanarayan Hariharaputran, Duc Vinh Le

**Affiliations:** Institute of High Performance Computing (IHPC), Agency for Science, Technology and Research (A*STAR), 1 Fusionopolis Way, Connexis, Singapore, 138634 Republic of Singapore

**Keywords:** Mathematics and computing, Physics

## Abstract

We simulated a pulsed direct current (DC) planar magnetron discharge using fluid model, solving for species continuity, momentum, and energy transfer equations, coupled with Poisson equation and Lorentz force for electromagnetism. Based on a validated DC magnetron model, an asymmetric bipolar potential waveform is applied at the cathode at 50–200 kHz frequency and 50–80% duty cycle. Our results show that pulsing leads to increased electron density and electron temperature, but decreased deposition rate over non-pulsed DC magnetron, trends consistent with those reported by experimental studies. Increasing pulse frequency increases electron temperature but reduces the electron density and deposition rate, whereas increasing duty cycle decreases both electron temperature and density but increases deposition rate. We found that the time-averaged electron density scales inversely with the frequency, and time-averaged discharge voltage magnitude scales with the duty cycle. Our results are readily applicable to modulated pulse power magnetron sputtering and can be extended to alternating current (AC) reactive sputtering processes.

## Introduction

Pulsed direct current planar magnetron (P-DCM) is frequently employed in reactive sputtering to deposit dielectric thin film such as Aluminium Scandium Nitride (AlScN)^1^ or Aluminium Nitride (AlN)^2^. In P-DCM, a bi-polar pulsed voltage is applied at a mid-frequency of 10–250 kHz^3^ leading to sputtering during the negative pulse and the discharging during the positive pulse. Advantages of P-DCM include a higher deposition rate compared to radio frequency (RF) sputtering^4^, a higher power compared to a non-pulsed DCM^5^ and reduced electrical arcing during sputtering^6^. Electrical arcing can occur due to accumulation of surface charges on the metal target, severely compromising the uniformity and quality of the deposited film^7^.

Using time-resolved Langmuir probe, Bradley et al*.*^8^ measured the time evolution of electron density and effective electron temperature of both DCM and P-DCM with duty cycle of 80% at the location near the substrate. The reported time-averaged electron density is $9.3\times {10}^{15}$ m^−3^, $8.4\times {10}^{15}$ m^−3^ for P-DCM at 50, 100 kHz greater than $7.1\times {10}^{15}$ m^−3^ for DCM, and the time-averaged electron temperatures as 4.2, 4.5 eV for P-DCM at 50, 100 kHz greater than 3.34 eV for DCM. Lee et al*.*^9^ reported a measurement of electron temperature of 3.06, 3.63, 5.32 eV for pulsed frequency of 75, 100, 250 kHz at duty cycle of 80%, respectively. Glocker^4^ compared direct current (DC) magnetron and 35 kHz alternating current (AC) magnetron at the same power and reported electron energies, ion densities and deposition rates of 3.2 eV, $6.4\times {10}^{16}$ m^−3^, 0.70 nm/s for AC, and 2.4 eV, $1.63\times {10}^{16}$ m^−3^, 0.82 nm/s for DC, respectively.

Lee et al*.*^10^ reported decreasing deposition rate at pulse frequencies less than 20 kHz, supported by similar results for vanadium oxide deposition at frequencies up to 350 kHz^11^. Deposition rates were generally found to increase with duty cycles^12,13^. Langmuir probe measurements of a 20 kHz P-DCM showed that increasing duty cycle from 10 to 90% under constant power leads to reduction in electron densities and temperatures^14^.

Due to operational challenges and material loss, computational modelling is an economic way to test and validate complex sputtering models^15^. Fluid models are among the simplest tools for modelling species in hydrodynamic equilibrium and find applications in non-pulsed DCM discharge^16^, high frequency pulsed DC discharge in nitrogen^17^ and capacitively couple plasma at RF frequency^18^. To improve model accuracy, hybrid numerical models, such as fluid model/Monte Carlo^19^ and particle-in-cell/Monte Carlo^20,21^, were proposed.

Despite the importance of P-DCM in reactive sputtering, there are to our knowledge scant report on numerical simulation. Currently, experimental studies using Langmuir probes for point-wise measurements are limited significantly in terms of spatial resolutions especially large gradients in terms of electron density, electron temperature, ion density and energy. Numerical simulations of P-DCM can be greatly advantageous for filling spatial information gaps and can provide rapid economical assessment of new pulsed sputtering designs.

In the present work, we model P-DCM using a fluid model in a two-dimensional axisymmetric P-DCM setup^16^, including an additional applied voltage wave form at the cathode whose frequency and duty cycle range 50–200 kHz and 50–80%, respectively. Specifically, we solve the continuity equations for electron, ion, neutral species, electron energy transfer equation, coupled with Poisson equation for discharge potential, and momentum transfer equations for drift–diffusion electron and ions fluxes. The magnetic field due to external permanent magnets is incorporated in the fluid model through the electron mobility tensor. Our simulation results are in qualitative agreement with experimental reports found in literature.

Although the fluid model is computationally efficient, it is less accurate under rarefied conditions where the mean free path of charged particles exceed the characteristic length of the discharge. Hence, the drift–diffusion approximation may not be valid under low working gas pressures, even though local collisions may be enhanced by focusing of electrons near the cathode^16^. Despite these limitations, our proposed approach is able to simulate the discharge in P-DCM and the results compare favorably with experimental measurements.

This paper is organized as follows. Details of the numerical method and simulation setup are provided in "Numerical method", the numerical result and discussion are provided in "Result and discussion". Finally, results are summarized in "Conclusions".

## Numerical method

### Governing equations

The governing equations of pulsed DC magnetron discharge for the fluid model^22^ describe four species, namely electron $\left(e\right)$, argon ion $\left({Ar}^{+}\right)$, excited argon $\left({Ar}^{*}\right)$ and neutral argon $\left(Ar\right)$, as follows:

1. Continuity equation for electron density and electron flux vector are
  1 $$\frac{\partial }{\partial t}\left({n}_{e}\right)+\nabla \cdot  {{\varvec{\Gamma}}}_{e}={R}_{e,}$$
  1a $${{\varvec{\Gamma}}}_{e}=-{n}_{e}\left({{\varvec{\mu}}}_{e}\cdot  {\varvec{E}}\right)-\nabla \left({{{\varvec{D}}}_{e}n}_{e}\right).$$
2. Electron energy density transfer equation and electron energy flux vector are
  2 $$\frac{\partial }{\partial t}\left({n}_{\varepsilon }\right)+\nabla \cdot  {{\varvec{\Gamma}}}_{\varepsilon }+{\varvec{E}}\cdot  {{\varvec{\Gamma}}}_{e}={R}_{\varepsilon },$$
  2a $${{\varvec{\Gamma}}}_{\varepsilon }=-{n}_{\varepsilon }\left({{\varvec{\mu}}}_{\varepsilon }\cdot  {\varvec{E}}\right)-\nabla \left({{\varvec{D}}}_{\varepsilon }{n}_{\varepsilon }\right).$$
3. Continuity equations for argon ion density and argon ion flux vector are
  3 $$\frac{\partial }{\partial t}\left({n}_{i}\right)+\nabla \cdot  {{\varvec{\Gamma}}}_{i}={R}_{i},$$
  3a $${{\varvec{\Gamma}}}_{i}={n}_{i}\left({{\varvec{\mu}}}_{i}\cdot  {\varvec{E}}\right)-\nabla \left({{{\varvec{D}}}_{i}n}_{i}\right).$$
4. Continuity equations for uncharged species (excited and neutral argons) densities are
  4 $$\frac{\partial }{\partial t}\left({n}_{m}\right)-\nabla \cdot  \left({{\varvec{D}}}_{m}\nabla {n}_{m}\right)={R}_{m},$$

where $\left\{{n}_{e}, {n}_{\varepsilon }, {n}_{i}, {n}_{m}\right\}$, $\left\{{R}_{e}, {R}_{\varepsilon }, {R}_{i}, {R}_{m}\right\}$, $\left\{{{\varvec{D}}}_{e}, {{\varvec{D}}}_{\varepsilon }, {{\varvec{D}}}_{i}, {{\varvec{D}}}_{m}\right\}$ and $\left\{{{\varvec{\mu}}}_{e}, {{\varvec{\mu}}}_{\varepsilon }, {{\varvec{\mu}}}_{i}, {{\varvec{\mu}}}_{m}\right\}$ are the densities $n$, reaction sources $R$, diffusivities ${\varvec{D}}$ and mobilities ${\varvec{\mu}}$, where subscripts represent electron $e$, electron energy $\varepsilon$, ionic $i$ and uncharged species $m$, respectively. Note that diffusivity ${\varvec{D}}$ and mobility ${\varvec{\mu}}$ can take the form of either tensor or scalar depending on the species. ${\varvec{E}}$ is the electric field.

The continuity equations (Eqs. 1–4) prescribe conservation of mass and energy, where the first and second terms on left hand side are the rate of change of and net flux of electron, electron energy, argon ion and neutral atom densities, respectively. Terms on the right-side are the respective reaction sources and sinks. Note that the conservation of electron energy (Eq. 2) includes an additional term for Joule heating or cooling of electrons which couples Eq. (1). The corresponding drift–diffusion approximation equations (Eqs. 1a, 2a, 3a) breaks down the transport fluxes of electron, electron energy and argon, respectively, as drift fluxes due to electric field and diffusion fluxes due to thermal field.

The Poisson and electric field equations are

5 $${\nabla }^{2}V=-\frac{e\left({n}_{i}-{n}_{e}\right)}{{{\varepsilon }_{0}\varepsilon }_{r}},$$

5a $${\varvec{E}}=-\nabla V.$$

where $e$ is the charge of electron, ${\varepsilon }_{0}$ is the permittivity of vacuum, ${\varepsilon }_{r}$ is the dielectric constant of argon, $\overline{\varepsilon }$ is the mean electron energy and $V$ is the plasma potential.

The driff–diffusion approximation of ion flux $\left({{\varvec{\Gamma}}}_{e}\right)$ and electron flux $\left({{\varvec{\Gamma}}}_{i}\right)$ can be derived from momentum transfer equation^22^ which reads

6 $$\frac{\partial }{\partial t}\left({n}_{s}{m}_{s}{{\varvec{u}}}_{s}\right)+\nabla \cdot  {n}_{s}{m}_{s}{{\varvec{u}}}_{s}{{{\varvec{u}}}_{s}}^{T}=-\nabla \cdot  {{\varvec{p}}}_{s}+{q}_{s}{n}_{s}\left({\varvec{E}}+{{\varvec{u}}}_{s}\times {\varvec{B}}\right)-{n}_{s}{m}_{s}{{\varvec{u}}}_{s}{v}_{m}$$

where subscript “*s*” is the type of particle (*s* = *e* for electron and *i* for ion, respectively), ${n}_{s}$ is the particle density, ${m}_{s}$ is the particle mass, ${{\varvec{u}}}_{s}$ is the drift velocity of particle, ${{\varvec{p}}}_{s}$ is the particle pressure tensor, ${q}_{s}$ is the particle change, ${\varvec{E}}$ is the electric field, ${\varvec{B}}$ is the magnetic field and ${v}_{m}$ is the momentum transfer frequency. The temporal and inertial force terms on the left-hand side of Eq. (6) are negligible due to the dominance of the terms on the right-hand side, which are pressure gradient, Lorentz force and particle collision force, respectively. This assumption is stood if a charged particle reacts instantaneously to a change of the electric field. The typical momentum transfer frequency for electrons is about 100 MHz. For ions, the characteristic momentum transfer frequency is only a few megahertz. In our simulation the pulsed frequency is in range of kilohertz which is smaller than the ion momentum transfer frequency.

The electron, argon ion and electron energy density source terms due to plasma reactions are

7 $${R}_{e,i}=\sum_{j=1}^{M}{k}_{j}{n}_{j}{n}_{e},$$

8 $${R}_{\varepsilon }=\sum_{j=1}^{M}{k}_{j}{n}_{j}{n}_{\varepsilon }\Delta {E}_{j}.$$

where the reaction rate constants are

9 $${k}_{j}=\gamma {\int }_{0}^{\infty }\varepsilon {\sigma }_{j}\left(\varepsilon \right)f\left(\varepsilon \right)d\varepsilon ,$$

which are based on Maxwellian electron energy distribution function (EEDF)

10 $$f\left(\varepsilon \right)={\overline{\varepsilon }}^{\left(-3/2\right)}{\beta }_{1}exp\left(-{\varepsilon \beta }_{2}/\overline{\varepsilon }\right).$$

where $\varepsilon$ is the individual electron energy, ${\beta }_{1}, {\beta }_{2}$ are the constants^22^, ${n}_{j}$ is the density of species participating the reaction j, $\Delta {E}_{j}$ is the energy lost in the reaction j, ${\sigma }_{j}$ is the collision cross section of reaction j and $M$ is the number of reactions, $\gamma ={\left(2e/{m}_{e}\right)}^{1/2}$ is a constant, $e$ and ${m}_{e}$ are the charge and mass of electron.

The mobilities of electron ${{\varvec{\mu}}}_{e}$, argon ion ${{\varvec{\mu}}}_{i}$ and energy ${{\varvec{\mu}}}_{\varepsilon }$ are respectively^22,23^,

11a $${{\varvec{\mu}}}_{e}^{-1}=\left[\begin{array}{ccc}\frac{1}{{\mu }_{dc}}& -{B}_{z}& 0\\ {B}_{z}& \frac{1}{{\mu }_{dc}}& {-B}_{r}\\ 0& {B}_{r}& \frac{1}{{\mu }_{dc}}\end{array}\right],$$

11b $${{\varvec{\mu}}}_{i}=\frac{4.411\times {10}^{19}}{{\left[1+{\left(7.721\times {10}^{-3}\left(\left|{\varvec{E}}\right|/{N}_{m}\right)\right)}^{1.5}\right]}^{0.33}{N}_{m}},$$

11c $${{\varvec{\mu}}}_{\varepsilon }={\left(\frac{5}{3}\right){\varvec{\mu}}}_{e},$$

where ${\mu }_{dc}$ is the electron mobility without the present of magnetic field, ${N}_{m}$ is the density of neutral argon and ${B}_{z}, {B}_{r}$ are the components of external magnetic field in cylindrical coordinates. The external magnetic field is independently calculated prior to the discharge simulation by solving the Maxwell equations for the case of static magnetic field. The electron mobility $\left({{\varvec{\mu}}}_{e}\right)$ accounts for the Lorentz force acting on electrons due to magnetic field. The mobility of ion $\left({{\varvec{\mu}}}_{i}\right)$ is the function of local electric field. The mobility of electron energy density $\left({{\varvec{\mu}}}_{\varepsilon }\right)$ obeys Einstein’s relation for a Maxwellian EEDF.

Lastly, the diffusion coefficients of electron ${{\varvec{D}}}_{e}$, ion ${{\varvec{D}}}_{i}$ and electron energy ${{\varvec{D}}}_{{\varvec{\varepsilon}}}$ are

12a $${{\varvec{D}}}_{e}={{\varvec{\mu}}}_{e}{T}_{e},$$

12b $${{\varvec{D}}}_{i}={{\varvec{\mu}}}_{{\varvec{i}}}{T}_{i},$$

12c $${{\varvec{D}}}_{\varepsilon }={{\varvec{\mu}}}_{\varepsilon }{T}_{e},$$

where ${T}_{e}=2\overline{\varepsilon }/3$ is the electron temperature and the ion temperature $\left({T}_{i}\right)$ is equal to the argon gas temperature. The elementary reactions for argon and their corresponding cross-sections are provided in Appendix A.

### Boundary conditions

The surface charge density $\left({\sigma }_{s}\right)$ accumulation condition is applied to dielectric wall boundaries as^22^

13 $$\frac{\partial {\sigma }_{s}}{\partial t}={\varvec{n}}\cdot  {\mathbf{J}}_{i}+{\varvec{n}}\cdot  {\mathbf{J}}_{e}$$

where ${\varvec{n}}\cdot  {\mathbf{J}}_{i}$ is the normal component of the total ion current density on the wall and ${\varvec{n}}\cdot  {\mathbf{J}}_{e}$ is the normal component of the total electron current density on the wall.

Under electrical conditions, the substrate anode is the reference potential, and the target cathode carries either a constant negative potential (DCM) or variable potential (P-DCM) where time-dependent waveforms can be applied.

Under discharge conditions, the electron flux $\left({{\varvec{\Gamma}}}_{e}\right)$ and electron energy flux $\left({{\varvec{\Gamma}}}_{\varepsilon }\right)$ along anode, dielectric wall and cathode boundaries with migration effects are^22^

14a $${\varvec{n}}\cdot  {{\varvec{\Gamma}}}_{e}=\frac{1}{2}{v}_{e,th}{n}_{e}+{n}_{e}\left({{\varvec{\mu}}}_{e}\cdot  {\varvec{E}}\right)\cdot  {\varvec{n}}-\gamma \left({{\varvec{\Gamma}}}_{i}\cdot  {\varvec{n}}\right)$$

14b $${\varvec{n}}\cdot  {{\varvec{\Gamma}}}_{\varepsilon }=\frac{5}{6}{v}_{e,th}{n}_{\varepsilon }+{n}_{\varepsilon }\left({{\varvec{\mu}}}_{\varepsilon }\cdot  {\varvec{E}}\right)\cdot  {\varvec{n}}-\gamma {\overline{\varepsilon }}_{i}\left({{\varvec{\Gamma}}}_{i}\cdot  {\varvec{n}}\right)$$

where $\gamma$ is the secondary electron emission coefficient (zero at the anode and dielectric wall) and ${\varvec{n}}$ is the outward normal vector of the boundary. The argon ion flux $\left({{\varvec{\Gamma}}}_{i}\right)$ and neutral species flux $\left({{\varvec{\Gamma}}}_{m}\right)$ along the same boundaries are^22^

15a $${\varvec{n}}\cdot  {{\varvec{\Gamma}}}_{i}=\frac{1}{2}{v}_{i,th}{n}_{i}-{n}_{i}\left({{\varvec{\mu}}}_{i}\cdot  {\varvec{E}}\right)\cdot  {\varvec{n}},$$

15b $${\varvec{n}}\cdot  {{\varvec{\Gamma}}}_{m}=\frac{1}{2}{v}_{m,th}{n}_{m}.$$

The species thermal velocity is defined by,

16 $${v}_{s,th}=\sqrt{\frac{8{k}_{b}{T}_{s}}{\pi {m}_{s}}}$$

where subscript “s” denotes *e* for electron, *i* for ion, *m* for neutral species and ${k}_{b}$ is the Boltzmann constant. Governing equations coupled with boundary conditions are solved using Finite Elements Method implemented on COMSOL Multiphysics (version 5.6).

### Simulation setup

**Figure 1:**
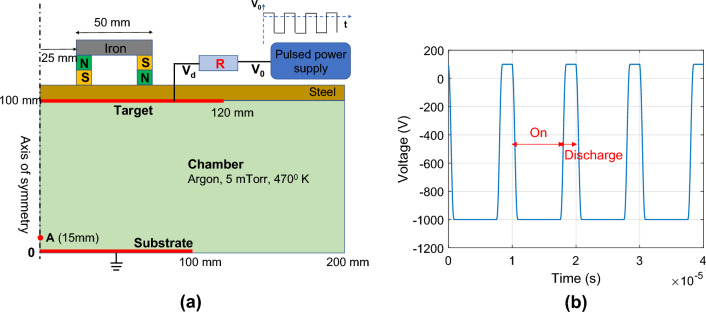
(**a**) Computational domain for an idealized two-dimensional axisymmetric pulsed DC magnetron discharge chamber and (**b**) Electric potential ${V}_{0}$ waveform as function of time $t$ at pulsed power supply. The applied frequency and duty cycle are 100 kHz and 80%, respectively.

The plasma chamber is modelled as a two-dimensional axisymmetric cylinder in (r, z) coordinates, containing argon gas held at pressure of 5 mTorr and temperature of 470 K (Fig. 1a). The substrate and target are modelled as flat circular discs with radii 120 mm and 100 mm respectively, spaced 100 mm apart in the axial (z) direction. The substrate as anode is grounded at reference potential, and the target as cathode is connected to a pulsed voltage signal ${V}_{0}\left(t\right)$ through a resistor of constant resistance $R$. This resistor is used to prevent arcing in a DC or pulsed DC magnetron discharges through a short-circuit, thus avoiding a feedback loop where the plasma densities become extremely high. The cathode potential at time $t$ follows the Ohmic relationship as

17 $${V}_{d}\left(t\right)={V}_{0}\left(t\right)-R{I}_{d}\left(t\right),$$

where ${I}_{d}\left(t\right)$ is the current flowing into electrode. The resistor effectively maintains a constant discharge power throughout fluctuating duty cycles.

An asymmetric bipolar potential waveform^24^ is applied at the target cathode. Figure 1b shows the waveform of pulsed voltage signal ${V}_{0}\left(t\right)$ at frequency of 100 kHz and duty cycle of 80%. Here we refer to “on time” as the duration of negative pulse at − 1000 V and “discharge time” as the duration of positive pulse at + 100 V.

Above the target and outside the computational domain, there are two concentric permanent magnets with opposite magnetic polarities and remanent flux density of 0.25 Tesla. The inner radius of magnet is 25 mm and distance between magnets is 50 mm.

**Figure 2:**
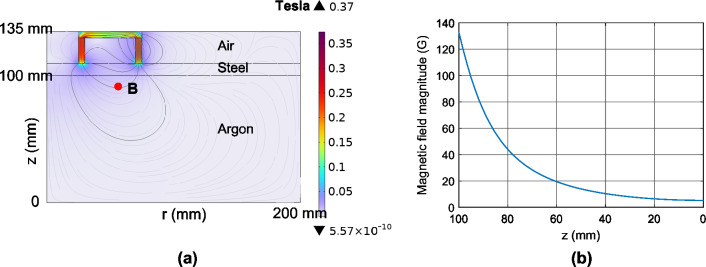
(**a**) Magnetic flux density magnitude and magnetic flux density streamline in the computational domain. (**b**) Magnetic flux density magnitude along z-direction at r = 60 mm.

Figure 2a shows the magnetic flux lines in the computational domain, which confine electrons through the Lorentz force^25^,

18 $${F}_{Lorentz}={q}_{e}{\varvec{E}}+{q}_{e}\left({v}_{e}\times {\varvec{B}}\right).$$

The out of plane Lorentz force vector results in a helical electron trajectory revolving around the chamber axis. The Lorentz force is at maximum when the local magnetic flux line is parallel to the substrate surface denoted by point B at radius r = 60 mm. Figure 2b shows the magnetic flux density magnitude along the z-direction at radius r = 60 mm, starting from 130 G on the target surface decaying to 5 G on the substrate.

## Result and discussion

We validate our model on the non-pulse DCM (Appendix B) before running it on pulsed mode as described in the following section.

### Pulse waveform

**Figure 3:**
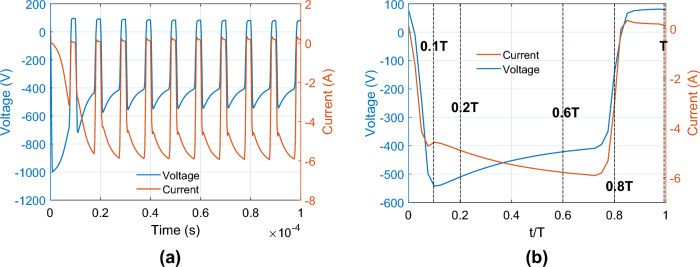
(**a**) Temporal evolution of discharge voltage ${V}_{d}\left(t\right)$ and discharge current ${I}_{d}\left(t\right)$ up to 0.1 ms. (**b**) Discharge current and discharge voltage in a stable period. The applied frequency and duty cycle are 100 kHz and 80%, respectively.

We ran pulse waveform for frequency of 100 kHz and duty cycle of 80%. At the cathode, the pulsed voltage waveform ${V}_{0}\left(t\right)$ is imposed through the resistor $\left(R=100\Omega \right)$. Figure 3a shows the temporal evolution of discharge voltage ${V}_{d}\left(t\right)$ and discharge current ${I}_{d}\left(t\right)$ over 10 pulse cycles. The initial discharge voltage is − 1000 V with no initial discharge current because the plasma has not been created yet. Soon plasma forms with gradual increase of discharge current magnitude from 0 up to 3 A and decrease of the discharge voltage magnitude from 1000 to 700 V as following Eq. (17).

During “discharge time”, both discharge voltage and current reverse polarity and decrease magnitude. Specifically, the discharge voltage reaches + 80 V which is maintained until the end of the cycle. The cycle then repeats, with discharge voltage amplitude decreasing and current amplitude increasing during ‘on time’, until stable waveforms are reached. The stable discharge period is obtained after four cycles.

Figure 3b shows a snapshot of the discharge current and discharge voltage within a stable cycle of P-DCM discharge. The discharge current magnitude peaks at − 5.9 A towards the end of “on time” (0.8 T) while the discharge voltage magnitude peaks at − 540 V at the beginning of “on time” (0.2 T).

### Electron density and potential field

**Figure 4:**
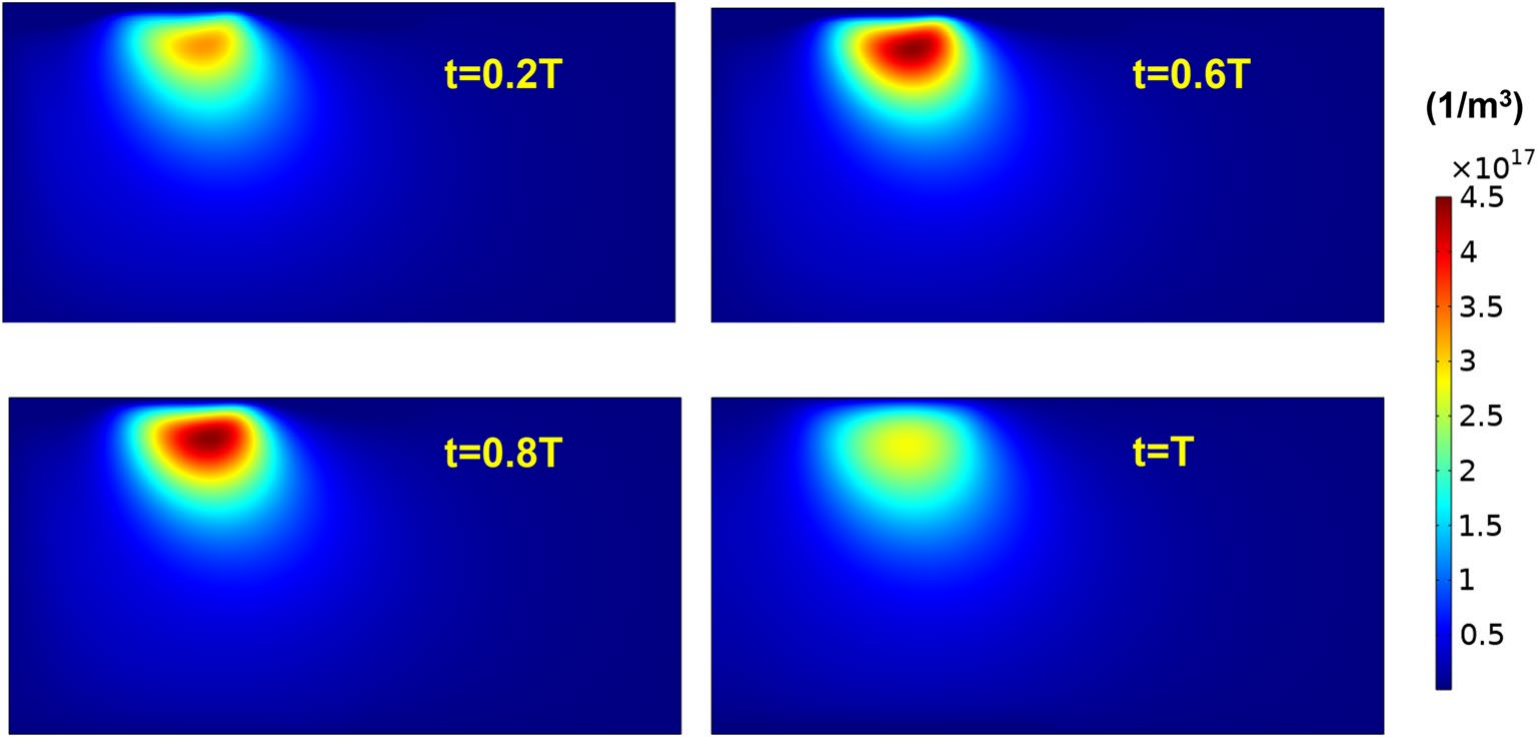
Snapshots of electron density distribution of pulsed DC magnetron at times t = 0.2, 0.6, 0.8 and 1.0 T. The applied frequency and duty cycle are 100 kHz and 80%, respectively.

For a given stable cycle of period T, Fig. 4 shows the electron density distribution at t = 0.2, 0.6, 0.8 and 1.0 T. Electrons accumulate near the region marked by point B in Fig. 2a at the onset of ‘on time’ (0.2 T) to a peak density of $4.5\times {10}^{17} {\mathrm{m}}^{-3}$ (0.6 and 0.8 T), followed by gradual decay till the end of the cycle (1 T). However, the plasma is still persistent in the “discharge time” which is also observed in the experiment^8,26,27^.

**Figure 5:**
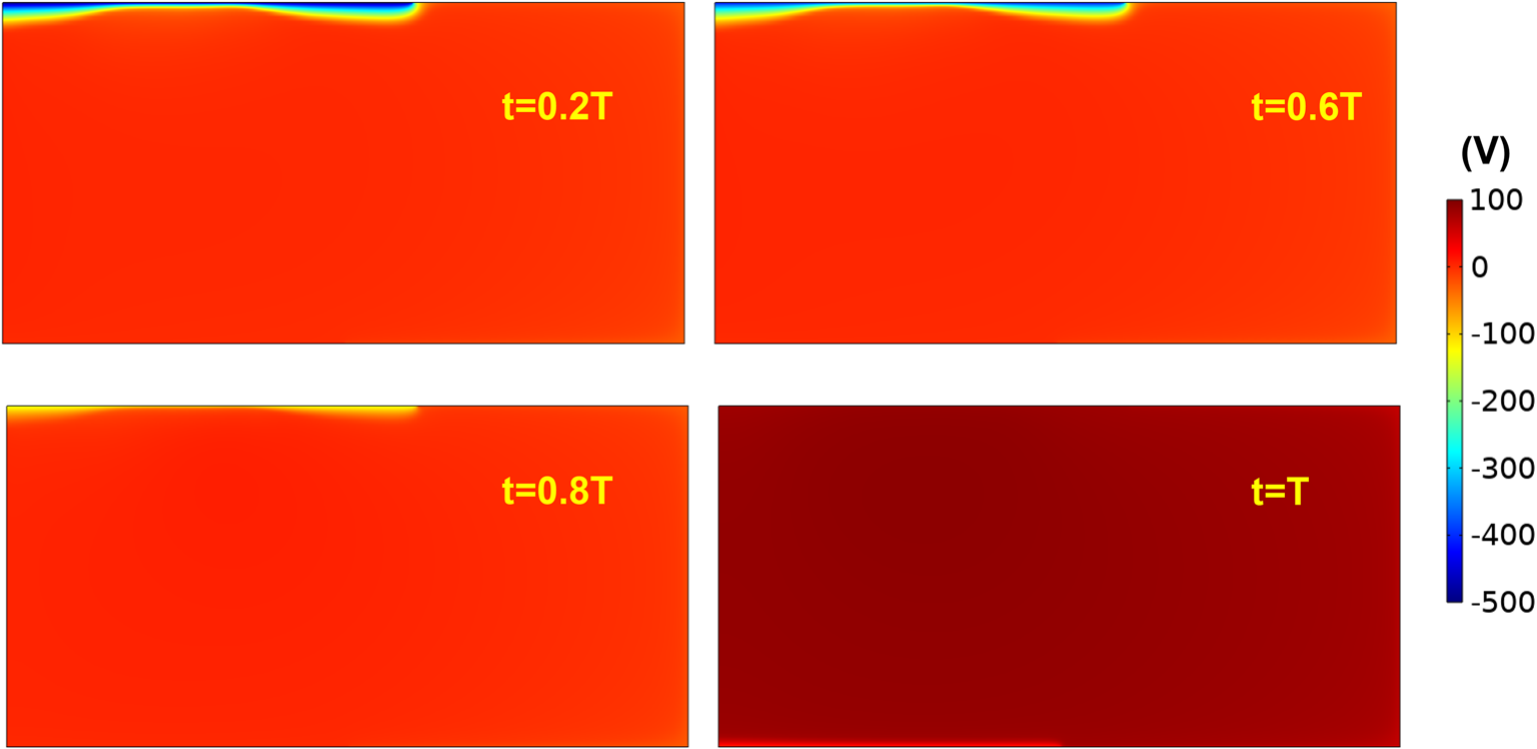
Snapshots of discharge electric potential distribution of pulsed DC magnetron at times t = 0.2, 0.6, 0.8 and 1.0 T. The applied frequency and duty cycle are 100 kHz and 80%, respectively.

Figure 5 shows the corresponding discharge electric potential distribution at times t = 0.2, 0.6, 0.8 and 1.0 T, the potential gradient is steepest near the cathode, except when polarity reverse, the steepest potential gradient is near the anode.

### Argon ion flux and electric field

In magnetron sputtering, the bombardment of energetic argon ions on target knocks out atoms to be transported to the substrate and deposit on its surface. Hence, the flux of energetic argon ions on target can affect the overall deposition rate and product film uniformity. On substrate, the flux of impacting energetic argon ion also plays a significant role in film quality.

**Figure 6:**
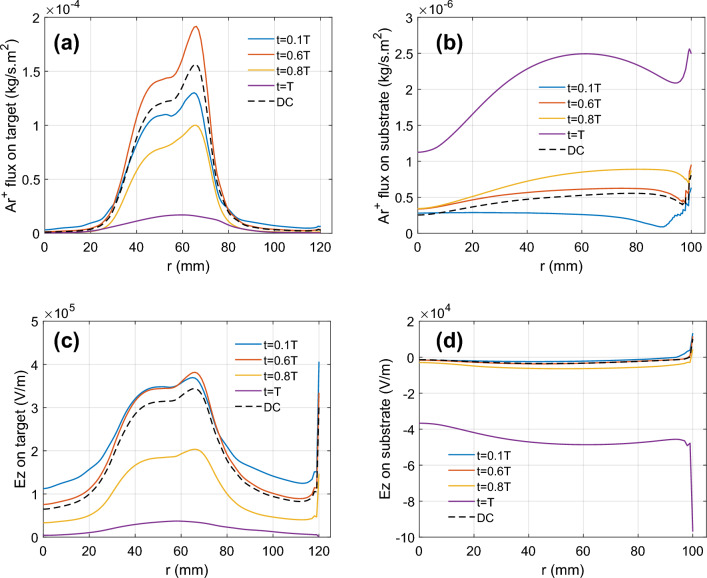
Argon ion (Ar^+^) flux on (**a**) target and (**b**) substrate vs. radial coordinate for pulsed DC magnetron plasma at times t = 0.1 T, 0.6 T, 0.8 T, 1 T and DC magnetron plasma (dashed line). Axial electric field (Ez) on (**c**) target and (**d**) substrate vs. radial coordinate. The applied frequency and duty cycle are 100 kHz and 80%, respectively.

Figure 6a,b show the argon ion normal fluxes on target and substrate for both pulsed and non-pulsed DC magnetron plasma at t = 0.1, 0.6, 0.8 and 1.0 T. We set the same time-averaged discharge power $\left({P}_{d}=\overline{{I }_{d}{V}_{d}}\right)$ for P-DCM as DCM at 1850 W. On target surface (Fig. 6a), the argon ion flux increases from 0.1 to maximum at 0.6 T and decreases thereafter till t = T, when the discharge voltage switches polarity to ${V}_{d}=+80 V$ and the flux decreases significantly to less than 10% of the peak value at t = 0.6 T (see also Fig. 3b). The peak P-DCM flux is greater than non-pulsed DC magnetron where the argon ion flux is at steady state. On substrate surface (Fig. 6b), the argon ion flux trends opposite in time to the previous target flux, where fluxes are weak during “on time” but increases sharply during the “discharge time”.

Figure 6c,d shows z-component electric field (Ez) on target and substrate for both pulsed and non-pulsed DC magnetron plasma at t = 0.1, 0.6, 0.8 and 1.0 T. On target surface (Fig. 6c), the P-DCM electric field is stronger than non-pulsed DC magnetron from t = 0.1 to 0.6 T and weaker during “discharge time” but notably, remains positive. This trend correlates to the earlier results (see Figs. 3b, 5). On substrate surface (Fig. 6d), the electric field is negative even for non-pulsed DC magnetron, but nevertheless, the argon ion flux towards the substrate is non-zero (see Fig. 6b). This observation agrees with the findings from other non-pulsed DC magnetron simulation^16^. Towards t = T, the electric field magnitude grows dramatically also in tandem with significant increase in argon ion flux (Fig. 6b).

### Pulsed frequency

The effect of pulsed frequency on deposition rate, time-averaged electron density $\left({\overline{n} }_{e}\right)$ and time-averaged electron temperature $\left({\overline{T} }_{e}\right)$ near the substrate are experimentally studied by several researchers^4,8–11^.

Here we summarize the empirical findings qualitatively as:

- ${\overline{T} }_{e}$ is greater in P-DCM than in DCM^4,8^.
- ${\overline{T} }_{e}$ increases with pulse frequency^8,9^.
- ${\overline{n} }_{e}$ is greater in P-DCM than in DCM^8^.

There are some discrepancies in reported ${\overline{n} }_{e}$ trends, however. For example, Bradley et al*.*^8^ reported a decrease of ${\overline{n} }_{e}$ from $9.3\times {10}^{15}$ to $8.4\times {10}^{15}$ m^−3^ when pulse frequency increases from 50 to 100 kHz. Lee et al*.*^9^ reported an increase from $8.77\times {10}^{10}$ to $1.02\times {10}^{11}$ cm^−3^ when pulsed frequency increases from 75 to 100 kHz.

**Figure 7:**
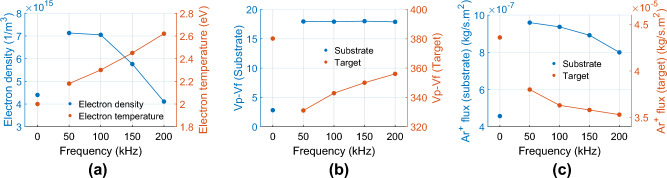
(**a**) Time-averaged electron density and electron temperature vs. frequency on the chamber axis at 15 mm above the substrate. Zero frequency denotes DC magnetron discharge. (**b**) Time-averaged difference of plasma potential (Vp) and float potential (Vf) (unit: Volt) on the chamber axis at substrate and target, and (**c**) time and spatial average of argon ion (Ar^+^) flux vs. frequency on either substrate or target. The duty cycle is 80%.

For model verification, we perform numerical simulations of P-DCM for pulsed frequencies 50, 100, 150 and 200 kHz with duty cycle 80% and time-averaged discharge power 1850 kW. Figure 7a shows, on the chamber axis at 15 mm above the substrate (point A in Fig. 1a), the time-averaged electron density and electron temperature for pulsed frequencies 50, 100, 150 and 200 kHz (0 Hz denotes non-pulsed DC magnetron discharge). The simulated time-averaged electron temperature for P-DCM increases linearly from 2.2 eV at 50 kHz to 2.6 eV at 200 kHz, greater than 2.0 eV for DCM. In contrast, the time-averaged electron density for P-DCM decreases linearly from $7.2\times {10}^{15}$ m^−3^ at 50 kHz to $4.1\times {10}^{15}$ m^−3^ at 200 kHz, also greater than that of DCM. Our results are therefore in agreement with the ${\overline{n} }_{e}$ trend as reported Bradley et al*.*^8^, rather than Lee et al*.*^9^.

The energy and flux of argon ion on substrate and target are important information in sputtering. Those information on target defines the sputtering yield and sputtered atoms flux from the target which determine the sputtering deposition rate. On substrate, the impingement of energetic argon ion flux can affect the film properties such as stress and roughness.

Figure 7b shows the time-averaged difference of plasma potential (Vp) and float potential (Vf) on substrate and target along the chamber axis for various applied frequencies. The frequency of zero is the DC magnetron discharge. Those potential differences are proportional to the energy of argon ion. Figure 7c shows the time and spatial average of argon ion flux on substrate and target.

Interestingly, on substrate, the potential difference (Vp–Vf) is independent to pulsed frequency, and the potential difference of P-DCM is 6.5 times bigger than that of DCM due to the change of potential polarities on electrodes. This means that the argon ion energy of P-DCM is higher than that of DCM which is consistent with the finding in Glocker’s work^4^. Besides, the argon ion flux on the substrate for P-DCM is also higher than that of DCM. Hence, the argon ion flux and energy impinging to the substrate in P-DCM is more significant than DCM. This can affect thin film properties such as stress or roughness when using P-DCM for sputtering.

On target, the potential difference of DCM is bigger than that of P-DCM. Hence, the argon ion bombarding to target of DCM is more energetic than that of P-DCM. Furthermore, more argon ion flux is seen in DCM case. As a result of that, the deposition rate using DCM will be bigger than that using P-DCM which is also found in the experimental work of Glocker^4^ and Dong^11^. Besides, the argon ion flux on target reduces when the pulsed frequency increases as shown in Fig. 7c. This results in the decreasing of deposition rate as rising of pulsed frequency which is consistent to the conclusion in literature^10,11^.

**Figure 8:**
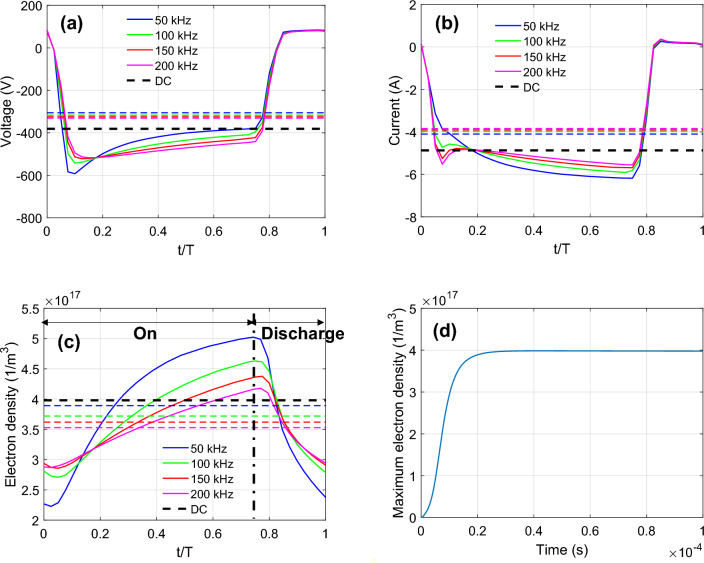
(**a**) Discharge voltage, (**b**) discharge current and (**c**) maximum electron density in the chamber for DC magnetron (black dashed line) and pulsed DC magnetron for various applied frequencies. Dashed lines refer to time-average results over a cycle. (**d**) Maximum electron density increases with time until steady state for non-pulsed DC magnetron simulation.

Within a stable cycle, the discharge voltage (Fig. 8a) and discharge current (Fig. 8b) for various pulsed frequencies follow similar profile as shown in Fig. 3b. The peak discharge voltage magnitude is − 600 V for 50 kHz (0.1 T) and decreases with frequency. Similarly, the peak discharge current magnitude is − 6.1 A for 50 kHz (0.7 T) and also decreases with frequency. We hypothesize that at lower frequencies, the pulsed discharge has more time to accumulate charges in the chamber during “on time”. Compared to P-DCM, DCM has greater time-averaged discharge voltage and current magnitudes.

Figure 8c shows how the maximum electron density in the chamber increases during “on time” followed by relaxation during “discharge time”, an observation consistent with experimental findings^26,27^. The highest electron density was observed at the lowest frequency, here 50 kHz which corresponds to the discharge current magnitude trend in Fig. 8b. Here, we note that the time-averaged electron density scales inversely with the cycle frequency (the frequency of non-pulsed DCM is zero). It takes approximately $2\times {10}^{-5}$ s for the plasma focus (denoted by maximum electron density) to reach steady state for non-pulsed DC magnetron simulation (Fig. 8d).

### Duty cycle

**Figure 9:**
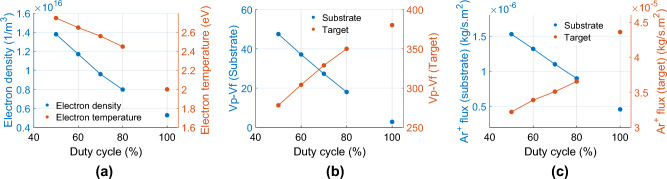
(**a**) Time-averaged electron density and electron temperature along the chamber axis at 70 mm away from target for duty cycle 50–80% (100% duty cycle for non-pulsed DCM). (**b**) Time-averaged difference between plasma potential (Vp) and float potential (Vf) (units: Volt) on the chamber axis at substrate and target, and (**c**) time and spatial average of argon ion flux on either substrate or target vs. duty cycle. The applied frequency is 150 kHz.

We study the effects of varying duty cycle from 50, 60, 70–80% for P-DCM (100% for non-pulsed DCM) at a fixed frequency of 150 kHz and time-averaged discharge power of 1850 W. Figure 9a shows that on the chamber axis at a distance of 70 mm away from the target, the time-averaged electron density and electron temperature decrease with increasing duty cycle, which is consistent with reported experimental trends^14^.

The time-averaged difference of plasma potential (Vp) and float potential (Vf) on substrate decreases with duty cycle and on target, increases with duty cycle (Fig. 9b). This dependence on duty cycle correlates with the argon ion energy and the time and spatial average of argon ion flux on both substrate and target (Fig. 9c). Since argon ion flux and energy on target increases with duty cycle, it can be inferred that deposition rates also increase with duty cycle, an implication in agreement with experimental reports^12,13^. Separately, the argon ion flux and energy on substrate decreases with increasing duty cycle, a result that may impact the quality of sputtered film.

**Figure 10:**
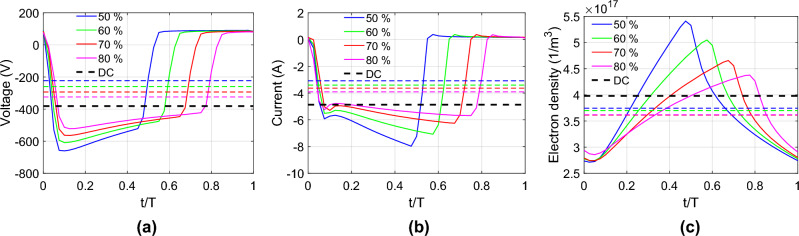
(**a**) Discharge voltage, (**b**) discharge current and (**c**) maximum electron density in the chamber for DC magnetron (black dashed line) and pulsed DC magnetron for duty cycle range 50–80% (black dashed line denote non-pulsed DCM). Dashed lines refer to time-average results over a cycle. The applied frequency is 150 kHz.

The discharge voltage (Fig. 10a) and discharge current (Fig. 10b) for P-DCM depends on the duty cycle, unlike non-pulsed DCM (black dashed line). Interestingly, we note that the magnitude of time-averaged discharge voltage scales with the duty cycle (the duty cycle of non-pulsed DCM is 100%). Compared to P- DCM, DCM has greater time-averaged discharge voltage and current magnitudes. However, the time-averaged electron density does not scale with duty cycle. Since discharge power is constant, the discharge current and voltage amplitudes are greater for lower duty cycle during “on-time”. This results in increased maximum electron density in the chamber with lower duty cycle (Fig. 10c).

## Conclusions

Using fluid model, we investigated numerically the effects of pulsed waveform with negative ‘on time’ and positive ‘discharge time’ potentials on pulsed direct current magnetron sputtering (P-DCM) compared to non-pulsed DCM. With our model validated through non-pulsed DCM simulation, we showed how the electrons are confined near the target by magnets and electric potentials vary during a pulse cycle, the argon ion fluxes to both target and substrate leading to deposition flux. Our simulation results are in qualitative agreement with empirical observations. On the question of whether time-averaged electron density should decrease^8^ or increase^9^ with pulse frequency, our numerical results corroborated with the former but only within the limited scope of this work.

Compared to non-pulsed DCM, we found that pulsing leads to increased electron density and electron temperature but decreased deposition rate. Increasing pulse frequency has the effect of increasing electron energy but reducing the electron density, argon flux on target and deposition rate. On the other hand, increasing duty cycle decreases both electron energy and density, but increases argon flux on target and deposition rate. Interestingly, we found that the time-averaged electron density scales inversely with the frequency, and time-averaged discharge voltage magnitude scales with the duty cycle. Our results are applicable to modulated pulse power magnetron sputtering^28^ and can be extended to alternating current (AC) reactive sputtering processes^3^.

Fluid model offers a simple yet powerful technique for simulating pulsed DC magnetron reactive sputtering for thin film deposition and the simulation tool gives much insight leading to improved operational heuristics. The current work estimates the ion flux and ion energy on the target used to calculate the sputtering yield and sputtered target atom flux, which can serve as input to a unified numerical framework of sputtering process^29^ using P-DCM. Future work could combine the pulsed DC magnetron discharge simulation with reactive gas and particle transportation models for numerical framework of sputtering process using P-DCM.

## Supplementary Information

Supplementary Information.

## Data Availability

The data used to support the findings of this study are available from the corresponding author upon request.
