# Supplementary material for: A fluid model of pulsed direct current planar magnetron discharge
Source: Sci Rep. 2023 Jun 3;13:9017. doi: 10.1038/s41598-023-36231-z (PMC10239496; doi:10.1038/s41598-023-36231-z)
Supplement: Supplementary file 1 — Supplementary Information. [file 41598_2023_36231_MOESM1_ESM.docx]

**Supplementary Material**

**A Appendix. Plasma reactions**

The elementary reactions for argon^1^ are shown in **Table A1.**

**Table A1. Elementary Argon plasma reactions**

| Index | Reaction | Type | ΔE (eV) | Constant |
| --- | --- | --- | --- | --- |
| 1 | $e+Ar\to e+Ar$ | Elastic collision | 0 |  |
| 2 | $e+Ar\to2e+{Ar}^{+}$ | Direct ionization | 15.8 |  |
| 3 | $e+Ar\to e+{Ar}^{*}$ | Excitation | 11.4 |  |
| 4 | $e+Ar\to e+Ar$ | Excitation | 13.1 |  |
| 5 | $e+{Ar}^{*}\to2e+{Ar}^{+}$ | Stepwise ionization | 4.4 |  |
| 6 | ${2Ar}^{*}\to e+{Ar}^{*}+{Ar}^{+}$ | Penning ionization |  | 6.2x10^-10^cm^3^s^-1^ |
| 7 | ${Ar}^{*}\to hv+Ar$ | Radiation |  | 1.0x107s^-1^ |

**Figure A1** shows the corresponding cross-section profiles^1^ with respect to electron energy.

**Figure A1. Electron cross sections for plasma reactions used in the model. Curve labels correspond to indices of corresponding processes in Table 1.**

**B Appendix. Model validation**

We validate our model by comparing our non-pulsed DCM results with those reported by Costin *et al*.^2^, also based on fluid model. Using the same geometry and discharge operating condition, we model the chamber as a cylinder with both radius and height equal to 26.95 mm, which carries argon gas at temperature of 350^0^ K and pressure of 20 mTorr (**Fig. B1a**). A constant potential of -550 V is applied to the lower target cathode of radius 16.5 mm; elsewhere, all other boundaries are grounded. Concentric magnets are positioned below the cathode, and they generate a magnetic field with field strength of 750 G on the cathode surface at the radial location of 9.5 mm, which focuses electrons as a spotlight about 6 mm away at radial position of 9.35 mm (**Fig. B1a**). Taking cross sections at radial position of 9.35 mm, we compare the electron density (n_e_) and argon ion density (n_i_) with Costin *et al*.^2^ and found good agreement in the results, with peak values of 2.5x10^16^ m^-3^ at z-location of 5 mm, as shown in **Fig. B1b**.

**

**Figure B1. (a) Modelled electron density distribution in indicated chamber geometry and discharge operating conditions. (b) Comparison between electron density (n_e_) and argon ion density (n_i_) along the z direction at radial position r = 9.35 mm with reported results from Costin *et al*.^2^, also based on fluid model.**

**References**

1 Rafatov, I., Bogdanov, E. A. & Kudryavtsev, A. A. On the accuracy and reliability of different fluid models of the direct current glow discharge. *Phys. Plasmas* **19**, 033502, doi:10.1063/1.3688875 (2012).

2 Costin, C., Marques, L., Popa, G. & Gousset, G. Two-dimensional fluid approach to the dc magnetron discharge. *Plasma Sources Sci. Technol.* **14**, 168, doi:10.1088/0963-0252/14/1/018 (2005).
